# Dataset of electric autonomous dial-a-ride instances with local energy communities and electricity tariffs

**DOI:** 10.1016/j.dib.2026.113063

**Published:** 2026-07-07

**Authors:** José Almeida, Steffen Limmer, João Soares, Maria Bresich, Günther Raidl, Zita Vale

**Affiliations:** a GECAD - Research Group on Intelligent Engineering and Computing for Advanced Innovation and Development LASI - Intelligent Systems Associate Laboratory ISEP, Polytechnic of Porto, Porto, Portugal; b HRI-EU - Honda Research Institute Europe, Offenbach, Germany; c Algorithmics and Complexity Group, TU Wien, Vienna, Austria

**Keywords:** Autonomous electric vehicles, Dial-a-ride problem, Electricity tariffs, Local energy communities, Porto taxi data

## Abstract

Shared autonomous electric vehicle (AEV) fleets offer significant potential for decarbonizing urban mobility, but their efficient operation requires jointly optimizing passenger routing and battery charging under real-world energy pricing constraints. The electric autonomous dial-a-ride problem (e-ADARP) formalizes this challenge, and its integration with local energy communities (LECs) introduces further complexity by coupling fleet scheduling decisions with time-varying community energy dynamics. This article presents a dataset of 20 benchmark instances for the e-ADARP integrated with LECs. The instances are derived from real-world taxi trip data from the city of Porto, Portugal, and incorporate energy data from two clustered LECs, each consisting of 10 prosumers with photovoltaic generation and individual load profiles. Each LEC is associated with a charging station located at the geographic centroid of its prosumers. The dataset comprises instances across 10 size configurations, ranging from 2 AEVs and 20 requests to 20 AEVs and 200 transportation requests, with two electricity tariff scenarios per configuration: a time-of-use tariff and a flat tariff, both incorporating mid-market rate pricing during periods of LEC overproduction. Each instance file encodes geographic coordinates of all nodes, time windows for pickups and dropoffs, vehicle parameters (battery capacity, energy consumption, minimum final state of charge), charging station characteristics, travel time matrices computed via OpenStreetMap shortest paths, and time-discretized energy profiles including community overproduction and electricity tariffs at 15-minute resolution over a 24-hour scheduling horizon. The dataset also includes the full LEC energy data workbook with individual prosumer load profiles, photovoltaic generation profiles, battery storage parameters, and electricity tariffs for both LECs, as well as the instance generation scripts, enabling researchers to develop alternative energy scenarios or modify community configurations. All files are publicly available in Zenodo.

Specifications Table

**Table:** 

Subject	Computer Sciences
Specific subject area	The instances are intended for combinatorial optimization, specifically autonomous electric vehicles (AEVs) routing and scheduling with local energy community (LEC) integration.
Type of data	Table (text files with structured numerical data) and a Table (.xlsx format); Processed; Generated/Simulated.
Data collection	The Porto taxi data from downtown Porto in Portugal together with the Portuguese electric mobility data were used to generate realistic pickup and dropoff points and candidate charging station locations. Prosumer load and photovoltaics (PV) generation profiles for local energy communities were taken from a publicly available dataset [1] and aggregated to 15-min resolution, in which realistic locations were generated for each user of the communities. Official Portuguese retail and feed-in tariffs were used for the grid transactions. All instances were then generated and normalized using custom Python and Julia scripts.
Data source location	City/Region: Porto metropolitan area, Portugal Coordinates: approximately 41.14°N to 41.25°N, 8.56°W to 8.69°W Institution: GECAD/ISEP, Polytechnic of Porto
Data accessibility	Repository name: Zenodo Data identification number: https://doi.org/10.5281/zenodo.20707207 Direct URL to data: https://zenodo.org/records/20707207 Instructions for accessing these data: The dataset is freely available for download under a Creative Commons Attribution 4.0 International (CC BY 4.0) license. No registration required. The repository contains 20 plain-text instance files (Instances.zip), the LEC energy data workbook (LEC_results_midPoint.xlsx), the LEC optimization output files used as input to the instance generator (results_LEC.zip), the instance generation script (generate_instances.jl), the LEC formation script (group_prosumers.py), and a README_v2.txt file describing the instance format and repository structure. The instance files and scripts are released under CC BY 4.0, explicit in the Zenodo repository [2]. The original Porto taxi trajectory data are publicly available at their original source under the authors' terms of use [3]. The electricity tariff data from SU Eletricidade are publicly available tariff schedules. Users should consult these original sources for redistribution and modification permissions.
Related research article	None. This is a standalone data article. The dataset was generated in the context of ongoing research on the e-ADARP with LEC integration; optimization models, algorithmic results, and experimental findings are outside the scope of this submission.

## 1 Value of the Data

• These instances represent the first publicly available benchmark set for the e-ADARP problem that integrates LEC energy overproduction and time-varying electricity tariffs (see Table 1 for a comparison with existing e-ADARP benchmarks) into the vehicle routing and charging optimization, filling a gap in the existing literature where e-ADARP benchmarks do not account for local distributed energy resources and different energy charging tariffs depending on local energy generation and demand, together with a charging cost component in the objective function that considers different time-varying electricity tariffs.

**Table 1:** Comparison of e-ADARP benchmark datasets.

Reference	Instance source	Max size	Tariff pricing	LEC Integration
[5]	Adapted Cordeau; San Francisco Uber	5 vehicles, 50 requests	None	No
[6]	Adapted Cordeau/Ropke; synthetic	260 vehicles, 5 200 requests	None	No
[7]	Adapted Cordeau/Ropke	8 vehicles, 96 requests	None	No
This work	Porto taxi data	20 vehicles, 200 requests	ToU + MMR	Yes
• The dataset serves multiple research communities: operations researchers can benchmark exact solvers on small instances (2–6 vehicles) and metaheuristics on larger ones (10–20 vehicles), while energy systems modelers can use the prosumer-level LEC workbook to study the economic impact of community energy sharing on fleet charging costs. Transportation planners can leverage real-world Porto geographic data to analyze routing behavior under realistic urban demand, and machine learning practitioners can use the scalable instance set to train and evaluate learning-based dispatch and charging agents.
• The practical applicability of the dataset lies in providing a common benchmark for transportation-energy optimization studies in which routing, charging, local renewable surplus, and electricity tariffs interact. Researchers can use the instances to construct baseline solutions with exact methods on the smaller cases, scalable heuristic or metaheuristic approaches on the larger cases, and learning-based dispatch or charging policies across the full set of 20 instances. The two tariff scenarios also allow users to quantify how tariff design and LEC overproduction affect charging decisions and operational costs under a common spatial and temporal setting. Thus, while this data article does not report solver benchmark results, it provides the standardized inputs required for future comparative studies.
• Two different cases are considered in the instances regarding the electricity tariffs: A time-of-use (ToU) tariff or alternatively flat tariffs are considered with a mid-market rate and are incorporated in each instance size allowing systematic investigation of how electricity pricing structures and charging infrastructure parameters affect optimal routing strategies and total operational costs.
• The instances are based on real geographic data from Porto (road network, taxi trajectories, user requests, and prosumer LEC locations), ensuring that travel times, distances, and energy profiles reflect plausible operational conditions rather than purely synthetic benchmarks. Time windows are synthetically generated to ensure full 24-hour coverage without peak-hour bias, and the deterministic single-day energy profiles represent a reproducible baseline. Extensions to stochastic or seasonal scenarios are identified as future work in the Limitations section.
• The scalable design provides a range of problem difficulties suitable for testing both exact methods on small instances and scalable heuristics on larger ones. It comprises 20 benchmark instance files across 10 size configurations from 2 vehicles and 20 requests to 20 vehicles and 200 requests, two tariff scenarios, and two LECs of 10 prosumers each.
• The accompanying LEC energy data provides individual prosumer-level profiles (load, PV generation, battery parameters, tariffs), enabling researchers to construct alternative community configurations, modify energy scenarios, or investigate sensitivity to prosumer composition, being able to generate their own instance data.
• The dataset supports a broad range of research applications, including benchmarking exact solvers (e.g., branch-and-cut, branch-and-price) on small instances and scalable metaheuristics (e.g., large neighborhood search) on larger ones. It also supports training and evaluating reinforcement learning or other learning-based agents for joint dispatch and charging scheduling, studying the sensitivity of routing and charging decisions to tariff design by comparing results across the time-of-use and flat tariff scenarios, and investigating the economic benefit of LEC integration by comparing charging costs under MMR and grid tariff conditions. A good extension would be the integration of vehicle-to-grid scenarios or seasonal photovoltaic variability using the prosumer-level energy data provided in the accompanying workbook.

## 2 Background

The dial-a-ride problem (DARP) [4] is a well-studied combinatorial optimization problem in which a fleet of vehicles must serve a set of user requests, each defined by a pickup and a dropoff location with associated time windows. The electric autonomous dial-a-ride problem (e-ADARP) [5] is a recent extension of this problem, which introduces autonomous electric vehicles (AEVs) with limited battery capacity, energy consumption modeling, and the need to schedule intermediate charging stops at dedicated charging stations.

However, existing benchmark instances for the e-ADARP do not incorporate energy community data, time-varying electricity tariffs, or realistic prosumer profiles. This dataset was generated to support research in e-ADARP integrating deterministic and metaheuristic optimization [6,7]. The LEC data originates from [8], a publicly available dataset of 100 residential prosumers with load, PV, and battery profiles. By making these instances publicly available as a standalone data article, we aim to provide the research community with a standardized testbed for evaluating competing approaches to this emerging problem variant. The instances were designed to capture the key features of the problem: real-world geographic routing on the Porto road network, heterogeneous energy supply from two distinct communities, and multiple electricity pricing scenarios reflecting current Portuguese retail tariff structures.

While the underlying data sources, namely the taxi trajectories, prosumer profiles, road network, and electricity tariffs, are individually publicly available, their integration into a unified benchmark that couples the transportation-energy optimization problem, with all parameters required to formulate the extended e-ADARP, constitutes the major contribution of this dataset, with Table 1 summarizing the key differences between the proposed dataset and existing e-ADARP benchmark sets from the literature.

## 3 Data Description

**Repository structure:** The Zenodo dataset repository contains the instance files organized by electricity tariff, and the source of LEC energy data as follows:

• ToU/ - Instances with ToU tariff (files are named r{numvehicles}-{numrequests}.txt, e.g., r2–20.txt through r20–200.txt).
• Flat/ - Instances with a flat tariff (files are named rF{numvehicles}-{numrequests}.txt, e.g., rF2–20.txt through rF20–200.txt).
• results_LEC.zip – LEC optimization output CSV files (balance profiles, MMR buy prices, and grid tariff buy prices for both LECs under both tariff scenarios) used as direct input to the instance generation script.
• generate_instances.jl – Julia entry-point script implementing the full instance generation pipeline. Running this script with the required input files reproduces all 20 instance files.
• group_prosumers.py – Python script implementing the LEC formation procedure. Running this script reproduces the prosumer clustering and charging station placement from the raw prosumer pool.
• LEC_results_midPoint.xlsx – Excel file containing the full LEC data used to generate the e-ADARP instances, including prosumer location and feeder connection, load profiles, PV generation, battery parameters, electricity tariffs, and grid connection limits for both LECs (19 sheets; see Table 4 for details).
• README_v2.txt – Plain-text file covering the repository structure and file contents, instance file format specification, software dependencies, and required package versions, step-by-step instructions for reproducing the dataset, approximate computational runtimes, license terms, and citation information. This is the recommended starting point for new users of the repository.

Table 2 summarizes the instance sizes in the dataset, regarding the total number of vehicles and requests, detailing the node IDs for each geographical location.

**Table 2:** Instance node configurations in the dataset.

Vehicles (K)	Requests (N)	Pickup nodes	Dropoff nodes	Origin depot node	Destination depot node	Charging station nodes
2	20	1–20	21–40	41	43	45–46
4	40	1–40	41–80	81	85	89–90
6	60	1–60	61–120	121	127	133–134
8	80	1–80	81–160	161	169	177–178
10	100	1–100	101–200	201	211	221–222
12	120	1–120	121–240	241	253	265–266
14	140	1–140	141–280	281	295	309–310
16	160	1–160	161–320	321	337	353–354
18	180	1–180	181–360	361	379	397–398
20	200	1–200	201–400	401	421	441–442

**Fig. 1:**
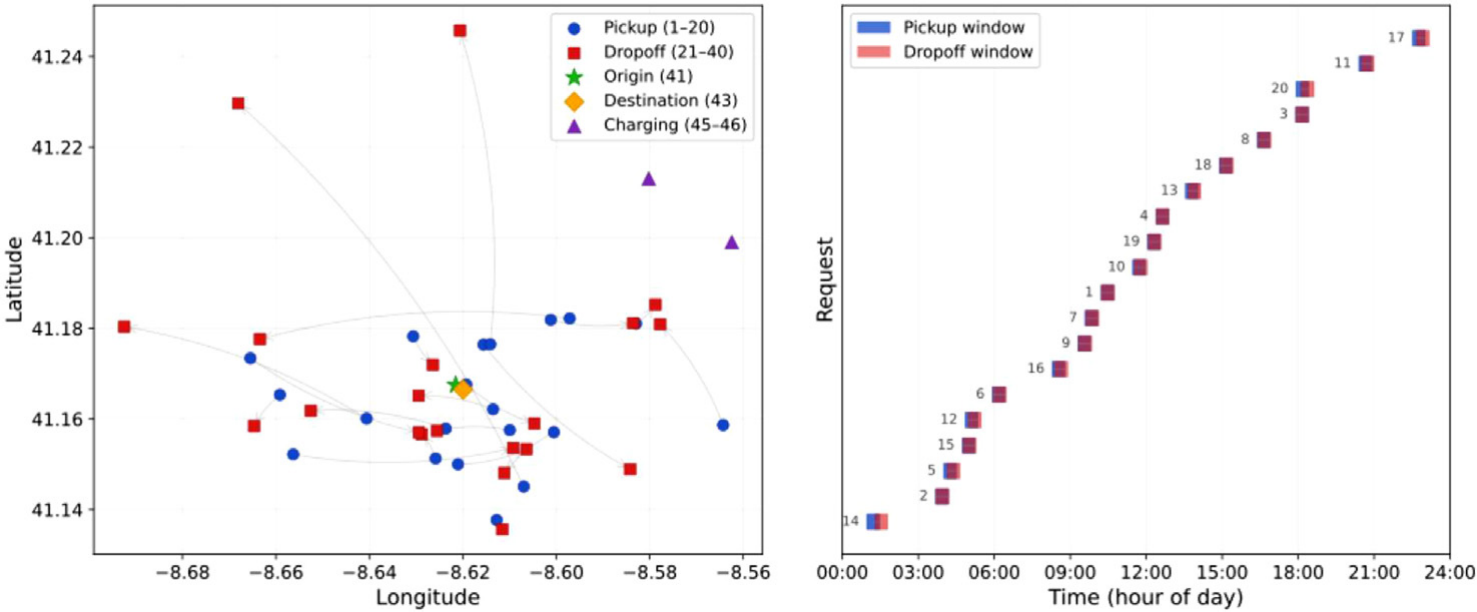
Instance file r2–20.txt: (left) geographic layout of pickup nodes (blue circles), dropoff nodes (red squares), origin depot (green star), destination depot (orange diamond), and LEC charging stations (purple triangles), with arrows connecting each pickup and dropoff pair; (right) time windows in minutes for each request, sorted by pickup time, showing pickup windows (blue) and dropoff windows (red).

Fig. 1 shows the geographic distribution and the time windows for the pickup and dropoff nodes, sorted by pickup time, for the smallest generated instance (r2–20). On the left are included the pickup and dropoff locations, origin and destination depots, which in this case share the same location, and the two charging stations located at the midpoint of the LECs. The node locations are identical across all tariff scenarios, as only the energy pricing parameters differ between instances of the same size.

**Instance file format:** Each instance file is a plain text file with the following structure. All coordinates are in the WGS84 geographic reference system (decimal degrees).

• **Header line:** #vehicles #users #origindepots #destinationdepots #stations #allowed visits #n. The header line shows the total number of vehicles and requests, the number of physical origin and destination depots, the number of charging stations, the maximum number of allowed visits per charging station (if greater than 1, additional virtual copies of each physical station are created in the node set to permit multiple visits during the scheduling horizon), and the total scheduling horizon in minutes.
• **Node block:** 〈node_id〉 〈lat〉 〈long〉 〈service duration〉 〈load〉 〈arr〉 〈dep〉. Each subsequent line following the header describes a node with its ID, geographic coordinates (latitude and longitude in decimal degrees), service duration in minutes (the time a vehicle spends stopped at that node), load change (the number of passengers boarding or alighting at that node), and the earliest and latest allowed service start times defining the node's time window. The load field represents the change in the number of passengers on board the vehicle upon visiting that node: a value of +1 at pickup nodes indicates one passenger boarding, and −1 at dropoff nodes indicates one passenger alighting. This ensures that the vehicle's cumulative occupancy never exceeds its passenger capacity. Nodes 1 to N are pickups of user requests (load = 1); nodes N + 1 to 2 N are dropoff nodes (load = −1); nodes 2N+1 to 2N+2 K are depot start/end pairs for each vehicle (load = 0); nodes 2N+2K+1 to 2N+2K+2 (load = 0) are the two LEC charging stations. In this case, N denotes the number of user requests, K denotes the number of AEVs.
• **Origin and destination depots:** <common origin depot id>; <common destination depot id>; <(artificial) origin depots id>; <(artificial) destination depots id>. Four lines specifying the physical origin and destination and virtual depots allowing the stay of multiple vehicles. Although all depot nodes share the same physical geographic location (the centroid of all request coordinates), the e-ADARP formulation requires each vehicle to have its own dedicated origin and destination node so that individual vehicle routes, departure times, and battery states can be tracked independently.
• **Charging station block:** 〈charging stations id〉. A single line indicating the physical charging station IDs (one per LEC).
• **Vehicle parameter block:** <users maximum ride time>; <vehicles capacity>; <vehicles initial battery level>; <vehicles battery capacities>; 〈minimum end battery ratio levels〉. This block details the vehicles’ parameters, as detailed in Table 6. The first line specifies the maximum time, in minutes, that each user can spend on board the AEV between pickup and dropoff nodes; the second parameter specifies the maximum number of passengers each vehicle can transport simultaneously. The third line represents the initial battery state-of-charge (SOC) in kWh at the start of the scheduling horizon. The following parameter shows the maximum battery capacity in kWh for each vehicle, and the final parameter is the minimum fraction of battery capacity that each vehicle must have remaining upon arrival at its destination depot.
• **Energy rates:** <recharging rates at charging station>; 〈vehicles discharging rate〉. Lines containing the maximum charging rate allowed in each charging station, and the energy consumed by each vehicle, both in kWh/min (values in Table 5, Table 6).
• **Cost weight factors:** <weight factors>; 〈cost factors〉. The first line details the standard weights used for the standard e-ADARP, while the second line contains the costs used in the current e-ADARP extension for the time travel and excess user ride time (€/min), which are applied when the objective function includes a monetary cost component alongside the charging costs from the LEC integration.
• **Energy overproduction profiles:** 〈 LECs overproduction〉. Two lines of 96 values representing the net energy overproduction (kWh) of LEC A and LEC B per 15-minute interval. These overproduction values can be bought by charging station operators at the MMR tariff and the remaining energy at ToU tariffs.
• **Electricity tariffs:** <Grid tariff>; <MMR tariff A>; 〈MMR tariff B〉. Three lines of 96 values: the base grid tariff (€/kWh), the effective purchase price for the charging station in LEC A (adjusted for community surplus), and the effective purchase price for the charging station in LEC B.
• **Travel time matrix: 〈**original travel times〉. A full (2*N + 2*K + 2) x (2*N + 2*K + 2) matrix of pairwise travel times in minutes, computed as the shortest-path distances on the OpenStreetMap road network divided by the average driving speed of 40 km/h. The original distances can be recovered by multiplying the travel times by 2/3 (i.e., $\frac{40\;km}{60min}$).

Table 3 provides a compact variable dictionary summarizing each field in the instance files, their units, meaning and the corresponding file given in the Zenodo repository.

**Table 3:** Variable dictionary for the instance file format: each field, its block location within the file, unit of measurement, physical meaning, and corresponding file in the Zenodo repository.

Field	Block	Unit	Meaning	Zenodo file
lat, lon	Node block	Decimal degrees (WGS84)	Geographic coordinates	Instances.zip
service duration	Node block	minutes	Time vehicle stops at node	Instances.zip
Load	Node block	passengers	Change in occupancy (+1 pickup, −1 dropoff)	Instances.zip
tw_start, tw_end	Node block	minutes	Earliest/latest service start	Instances.zip
max ride time	Vehicle block	minutes	Maximum user on-board time	Instances.zip
battery capacity	Vehicle block	kWh	Maximum battery energy	Instances.zip
initial battery	Vehicle block	kWh	Battery level at start	Instances.zip
min final SOC	Vehicle block	ratio [0,1]	Minimum battery fraction at destination depot	Instances.zip
charging speed	Energy rates	kWh/min	Maximum charging rate per CS	Instances.zip
consumption rate	Energy rates	kWh/min	Energy consumed per minute of travel	Instances.zip
Overproduction	Energy profiles	kWh	LEC surplus per 15-min period	results_LEC.zip
grid tariff	Electricity tariffs	€/kWh	Grid buy price per 15-min period	results_LEC.zip
MMR price	Electricity tariffs	€/kWh	Mid-market rate per 15-min period	results_LEC.zip
travel time	Matrix	minutes	Shortest-path travel time between nodes	Instances.zip

**Fig. 2:**
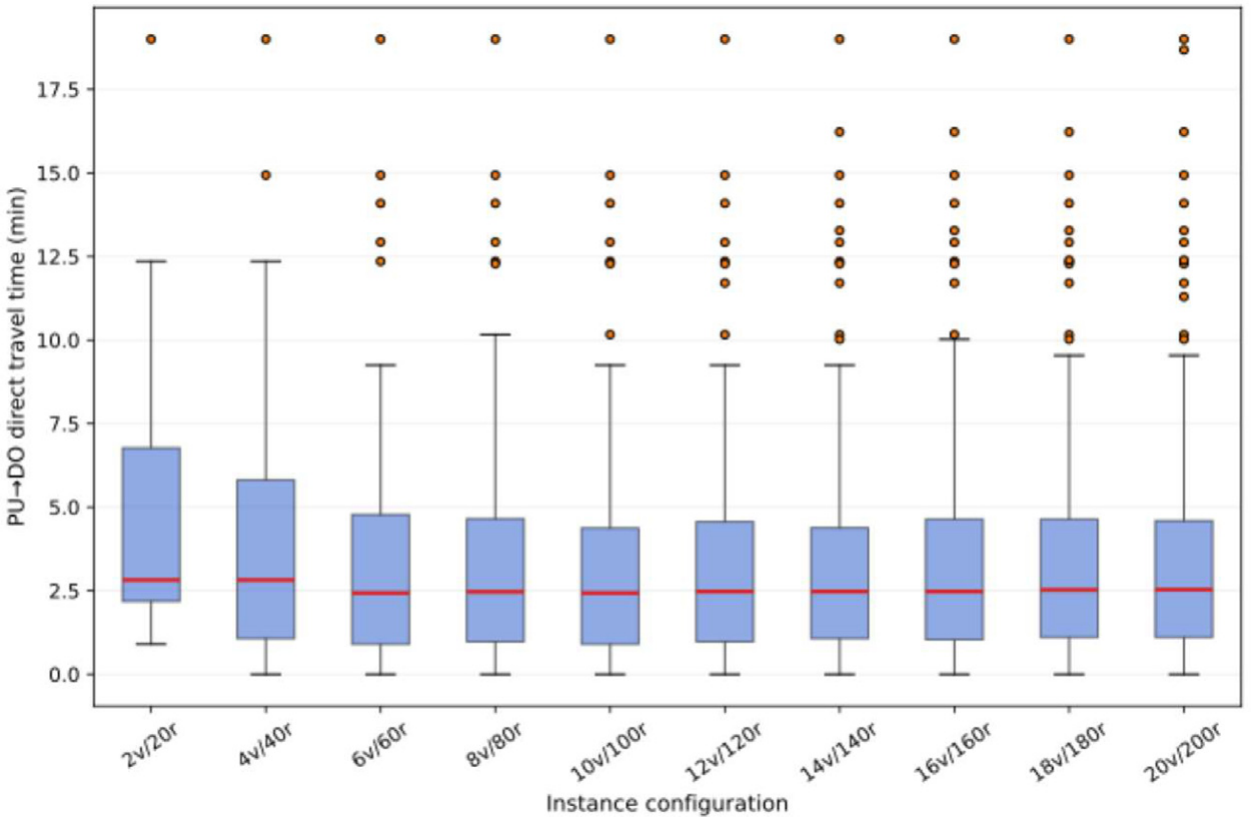
Distribution of direct pickup-to-dropoff travel times (minutes) across all 10 instance configurations, from r2–20 (2 vehicles, 20 requests) to r20–200 (20 vehicles, 200 requests), computed from travel time matrices in Instances.zip.

Fig. 2 shows the distribution of the travel times from the pickup to the dropoff nodes across all instance configurations. The configurations exhibit a similar travel-time pattern, with the median for each around 2.5 min. The maximum travel time is approximately the same across all instance sizes, since each larger instance includes all trips from the smaller ones. However, as the number of requests increases, the interquartile range widens and more trips with longer travel times appear, increasing the density of outliers in the upper range. This is expected, since larger instances draw from a broader pool of taxi trajectories, progressively including longer-distance trips that are not present in the smaller configurations.

Listing 1 presents an annotated excerpt from instance file r2–20.txt illustrating the structure of each block. Ellipses indicate omitted lines.



**Listing 1.** Annotated excerpt from instance file r2–20.txt (Instances.zip, Zenodo). Each line is shown with an inline annotation (←) identifying its block, field meaning, and units. Ellipses indicate omitted lines.

**LEC data:** The Excel workbook (LEC_results_midPoint.xlsx) provides the complete LEC data from which the e-ADARP instances’ energy parameters were derived. It contains 19 sheets organized by LEC and data type. Table 4 describes each sheet.

**Table 4:** Contents of the LEC community data workbook (LEC_results_midPoint.xlsx). Sheets prefixed A_ and B_ refer to LEC A and LEC B respectively. Time-series sheets contain 96 rows (one per 15-minute interval) and 10 columns (one per prosumer).

Sheet name	Dimensions	Description
Members_A / Members_B	10 × 4	Prosumer index, electrical feeder ID, latitude, and longitude for each prosumer
Stations	2 × 7	Charging station metadata: LEC label, station ID, coordinates, outlet power (kW), address
A_Load / B_Load	96 × 10	Electricity load consumption profiles (kW) per prosumer per 15-min period
A_PV / B_PV	96 × 10	Photovoltaic generation profiles (kW) per prosumer per 15-min period
A_Bat / B_Bat	7 × 10	Battery storage parameters: model ID, capacity (kWh), max charge/discharge rate (kW), round-trip efficiency, initial and final SOC (kWh)
A_Buy_price_ToU / B_ Buy_price_ToU	96 × 1	Electricity purchase price (€/kWh) under the time-of-use tariff
A_Buy_price_Flat / B_Buy_price_Flat	96 × 1	Electricity purchase price (€/kWh) under the flat tariff
A_Sell_price_ToU / B_Sell_price_ToU	96 × 1	Electricity feed-in (sell) price (€/kWh) under the time-of-use tariff
A_Sell_price_Flat / B_Sell_price_Flat	96 × 1	Electricity feed-in (sell) price (€/kWh) under the flat tariff
A_Limits / B_Limits	4 × 10	Grid connection limits: contracted buy/sell power (kW), fixed costs (€/day), and installed PV capacity (kWp)

**Fig. 3:**
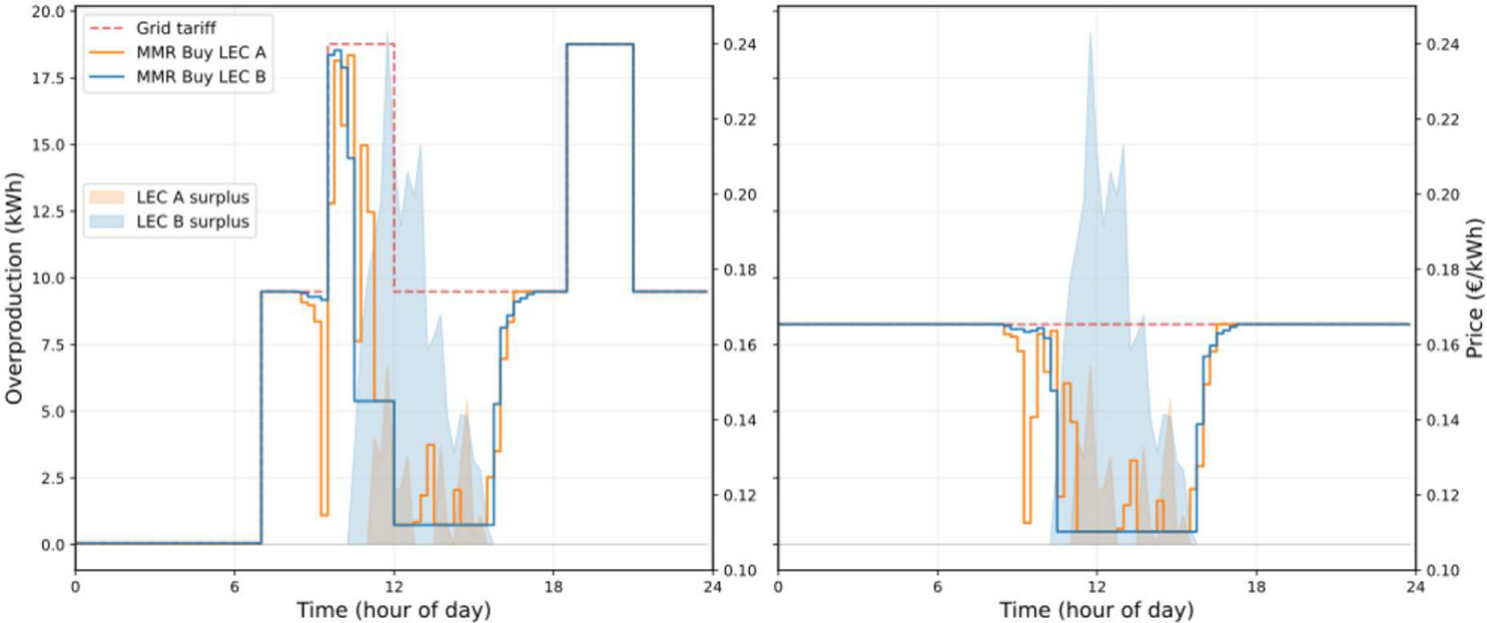
Comparison of the electricity tariff scenarios: (left) Time-of-Use, and (right) Flat, for LEC A and LEC B at 15-minute resolution (96 periods over 24 h). Shaded areas show LEC overproduction (left axis) in kWh; lines show grid tariff and the mid-market rate prices generated by computing LEC transactions, in €/kWh. During overproduction hours, the effective price drops below the grid tariff, creating charging cost incentives. Energy profiles from results_LEC.zip; tariff data from A_Buy_price_ToU and B_Buy_price_ToU sheets in LEC_results_midPoint.xlsx.

Each of the two LECs consists of 10 prosumers selected from a pool of 100 residential prosumers [8]. The prosumer data include 96-period load consumption profiles, photovoltaic generation profiles, battery storage parameters (model, capacity in kWh, charge/discharge rates, efficiency), and contracted power limits. Community energy overproduction at each time period is computed as aggregate PV generation minus aggregate load consumption across all LEC members, with only net surplus energy available for AEV charging. The prosumers in LEC A have installed PV capacities ranging from 0 to 6 kWp, while all prosumers in LEC B have nonzero PV installations, which explains the substantially higher overproduction in LEC B, as shown by the shaded areas in Fig. 3. Battery storage capacities range from 0 to 17.2 kWh for LEC A members and from 6.4 to 12.9 kWh for LEC B members.

Table 5 shows the parameters for the charging stations, located at the geographic centroid of each LEC, detailing the coordinates and outlet power. It also presents the charging speed associated with each charging station, meaning the amount of energy it supplies the AEVs in each minute, so for a 15-minute time slot the maximum amount of energy supplied is 5.51 kWh.

**Table 5:** Charging station parameters, from Stations sheet of LEC_results_midPoint.xlsx.

Parameter	LEC A	LEC B
CS location	Centroid (41.213°N, 8.580°W)	Centroid (41.199°N, 8.562°W)
CS outlet power	22 kW	22 kW
Charging speed (standard)	0.367 kWh/min	0.367 kWh/min
Peak overproduction	≈6.7 kWh (period 46)	≈19.2 kWh (period 47)

The considered AEV and e-ADARP parameters for the generated instances are detailed in Table 6. The table shows each vehicle's battery and passenger capacity, its energy consumption per minute at an average velocity of 40 km/h (1.5min/km, a typical value in urban areas), and the minimum battery state-of-charge (SOC) required at the end of the scheduling horizon. The table also describes the considered service time at each node, the maximum user ride time for each user request, the time window width, and the planning horizon, which in this case is a full day.

**Table 6:** Autonomous electric vehicle and e-ADARP parameters (based on Tesla Model 3 RWD), encoded in the vehicle parameter block of each instance file in Instances.zip.

Parameter	Value
Battery capacity	57.5 kWh
Passenger capacity	4
Energy consumption	0.09 kWh/min (at 40 km/h)
Minimum final SOC	70%
Average driving speed	40 km/h (1.5 min/km)
Service time per stop	3 min
Maximum ride time	60 min
Pickup and dropoff time window width	30 min
Planning horizon	1440 min (24 h)

## 4 Experimental Design, Materials and Methods

**Fig. 4:**
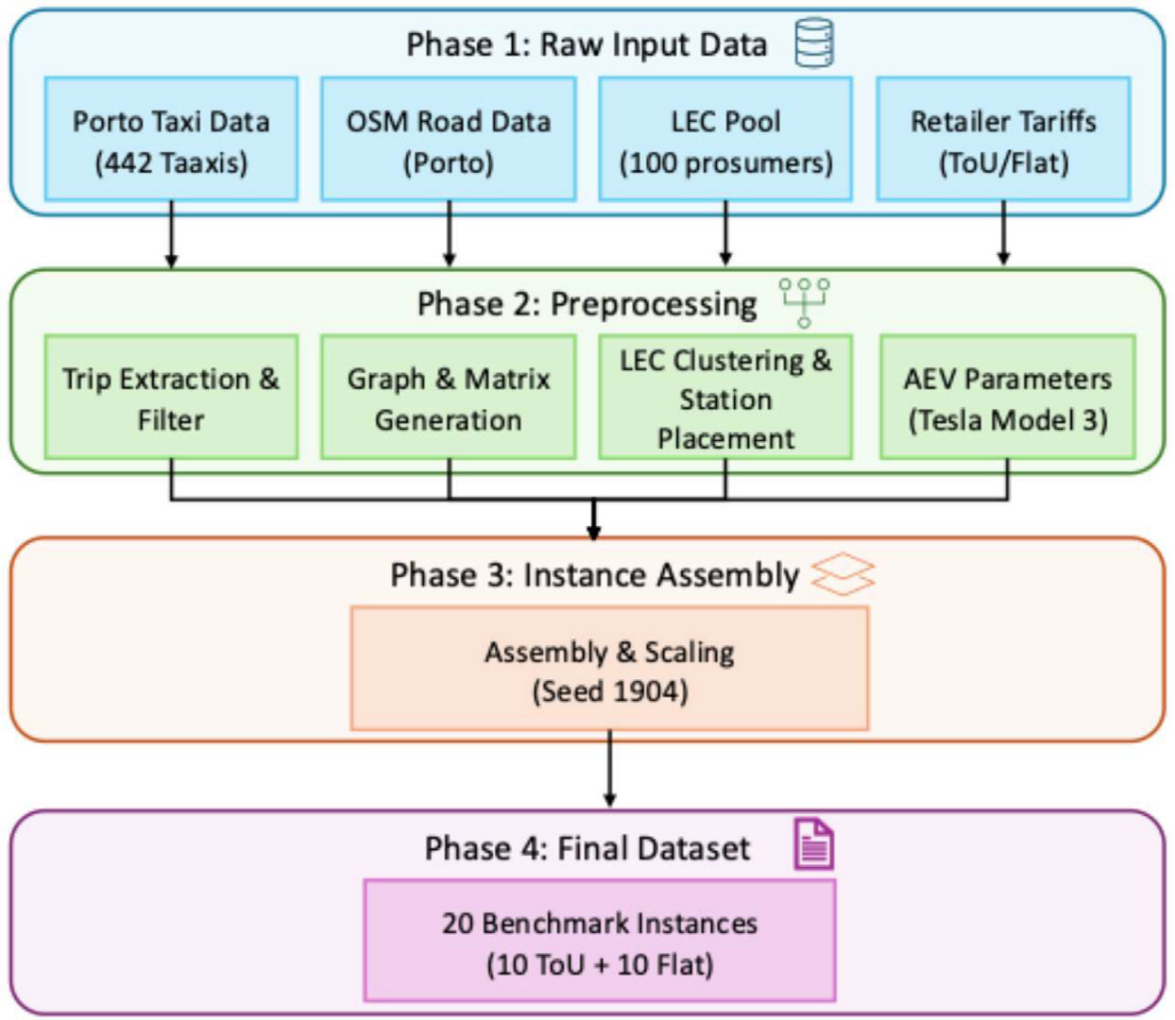
Dataset generation pipeline from raw input data to final e-ADARP benchmark instances. The workflow integrates Porto taxi trajectories, OpenStreetMap road data, LEC prosumer profiles, electricity tariffs, and vehicle parameters. The preprocessing stage includes trip extraction and filtering, graph and travel-time matrix generation, LEC clustering with charging-station placement, and PV/load and tariff processing. The final assembly stage generates 20 plain-text instances, corresponding to 10 size configurations under ToU and flat tariff scenarios.

The complete dataset generation pipeline is shown in Fig. 4. The dataset generation starts with four raw input sources: Porto taxi trajectories, OpenStreetMap road network data, the prosumer pool used to form the LECs, and Portuguese electricity tariffs. These inputs go through a preprocessing phase that performs trip extraction and filtering, graph and travel-time matrix generation, LEC formation via prosumer clustering, charging station placement (group_prosumers.py), and the inclusion of AEV parameters from the Tesla Model 3. The final assembly stage (generate_instances.jl) generates the output dataset with 20 plain-text instances, corresponding to 10 size configurations under ToU and flat tariff scenarios (Instances.zip in Zenodo).

Table 7 shows the software versions used to support reproducibility of the road-network extraction, graph processing, tabular data handling, and instance generation steps. This is particularly relevant for OSMnx and NetworkX, since changes in road-network extraction or graph-processing libraries may slightly affect graph topology, route availability, and shortest-path travel times. The fixed random seed of 1904 was used for deterministic time-window generation. Regarding computational requirements, the most time-consuming step is the pairwise shortest-path travel time matrix computation, which scales quadratically with the number of nodes. On a workstation with 36 GB of RAM and a 12-core CPU at 4.05 GHz, generating the smallest instance (r2–20, 46 nodes) takes approximately 1 to 2 min, while the largest instance (r20–200, 442 nodes) takes approximately 30 to 45 min. A standard machine with the minimum 8 GB of RAM is enough to run the pipeline. The OSMnx road network download requires an internet connection, and runtime may vary depending on server response times.

**Table 7:** Software environment used for dataset generation.

Component	Version	Role
Python	3.12	LEC preprocessing, data handling, and support scripts
OSMnx	2.10	OpenStreetMap road-network extraction and routing support
NetworkX	3.6.1	Graph processing and shortest-path operations
Pandas	2.3.3	Tabular data processing
NumPy	2.3.5	Numerical processing
openpyxl	3.1.5	Excel workbook reading
matplotlib	3.10.8	Figure generation
Julia	1.12.4	Instance assembly and final file generation
CSV.jl	0.10.15	CSV file reading/writing in Julia
DataFrames.jl	1.8.1	Tabular data handling in Julia
XLSX.jl	0.10.4	Excel workbook handling in Julia
PyCall.jl	1.96.4	Python-Julia interoperability
Random seed	1904	Deterministic time-window generation

After generation, the published instance files were checked for structural, temporal, spatial, and energy-data consistency. A set of consistency checks was performed on all generated instance files. These verified that files follow the expected plain-text structure and that node counts match the header information. Travel-time matrices were checked for dimensions compatible with the corresponding node sets, and all travel-time values were confirmed to be finite and non-negative. Pickup and dropoff time windows were verified to lie within the 1440-minute planning horizon. Finally, all LEC overproduction and tariff vectors were checked to contain exactly 96 values, corresponding to the 15-minute discretization of the 24-hour horizon. These checks were performed on the generated files and do not require solving the e-ADARP instances.

**Problem definition and LEC extension:** The e-ADARP [5] is defined on a complete graph G=(V,A), where the vertex set V=N∪O∪F∪C comprises pickup nodes P={1,…,n}, dropoff nodes D={n+1,…,2n}, origin depots O, destination depots F, and charging stations C. A set of K homogeneous AEVs must serve all n user requests, where each request i∈P has a corresponding dropoff at n+i∈D.

The e-ADARP minimizes a weighted combination of total travel time and total excess user ride time. For each request i, the excess ride time for request $R_{i}$ is defined as:

$$R_{i}=T_{n+i}^{k}-T_{i}^{k}-d_{i}-t_{i,n+i}$$

where $T_{i}^{k}$ represents service time for each vehicle k in each node i, $d_{i}$ is the service duration and $t_{i,n+i}$ represents the travel time from a pickup node i to a dropoff node n+i. Constraints enforce that each request is served exactly once by the same vehicle (pickup and dropoff), time windows are respected, vehicle capacity is not exceeded, maximum user ride times are observed, and battery levels remain feasible throughout each route.

The instances in this dataset extend the standard e-ADARP by introducing a charging cost component into the objective function. The scheduling horizon T is discretized into 96 time periods of 15 min each. At each period t, the energy charged by any vehicle at a LEC charging station is split into two components: energy purchased at the MMR, limited by the community's available overproduction $OLEC_{\left(t\right)}$, and the remainder purchased at the grid tariff (ToU or flat). The full objective thus becomes a weighted sum of charging costs, excess user ride time costs, and travel costs.

**User request generation:** User requests for the e-ADARP were derived from the Porto taxi trajectory dataset, originally published for the ECML/PKDD 2015 competition [3]. This dataset contains GPS traces of 442 taxis operating in Porto, Portugal, over a 12-month period. The first coordinate of each trip polyline defines the pickup location, and the last coordinate defines the dropoff location. Trips with disconnected routing (e.g., trip T115, whose dropoff has no road connection to the depot area) were excluded. No additional exclusion criteria were applied to the taxi trajectories beyond removing trips with disconnected routing. Trips with malformed or empty polyline fields were not present in the selected subset of centrally dispatched (“CALL_TYPE = A”) trips used for instance generation.

For each instance size N, the first N eligible trips were selected. Pickup time windows were generated uniformly at random over the planning horizon (with a 30-minute buffer from each end), each with a width of 30 min. Dropoff time windows were computed by adding the direct travel time from pickup to delivery to the pickup time window bounds. A fixed random seed of 1904 and Julia's MersenneTwister random number generator were used to ensure reproducibility of time window generation across all instance sizes. The Porto taxi dataset contains actual trip timestamps that reflect real demand patterns. However, to ensure full 24-hour coverage across all instance sizes and avoid introducing a temporal bias, uniform sampling was deliberately chosen, resulting in a more uniform distribution of user requests over time than would be observed in operational data. Researchers intending to study algorithms in applied operational settings may examine the raw taxi data in the original dataset [3] and construct temporally realistic alternatives based on it.

**Road network and travel time computation:** The Porto road network was downloaded from OpenStreetMap using the OSMnx Python library [9]. A bounding box was computed from the extreme coordinates of all request locations, and the driving network within this region was extracted. Pairwise shortest-path distances were computed using Dijkstra’s algorithm [10] with edge length (meters) as weight, then converted to kilometers. Travel times in minutes were then obtained by dividing distances by the driving speed (40/60 km/min average urban speed). For node pairs where no shortest paths exist in the directed road network, an undirected view of the graph is used as a fallback. If no path exists in the undirected graph either, the haversine great-circle distance is used as a geometric approximation. No such fallback cases were encountered in the published instances. Energy consumption per arc is computed as β = β’ × t’, where β’ = 0.09 kWh/min is the vehicle consumption rate and t’ is the travel time.

**LEC formation:** The two LECs were formed from a pool of 100 residential prosumers with known geographic locations and electrical feeder assignments. A compatibility-constrained clustering algorithm was used. Two prosumers are considered compatible if they are within 2000 m of each other OR connected to the same electrical feeder. The corresponding prosumer profiles were directly derived from [8] without any modification, as the source dataset contains no missing data.

For each LEC, a virtual charging station was placed at the geographic centroid (mean latitude and longitude) of its members. This placement simulates a community-owned charging facility that would be centrally accessible to all prosumers.

**Energy profile computation:** Each prosumer u in a LEC L has a forecasted load demand of $p_{\left(u,t\right)}^{\text{load}}$ and a PV generation given by $p_{\left(u,t\right)}^{\text{PV}}$ at each time step t. The net balance of each user is given as $\lambda_{\left(u,t\right)}=\;p_{\left(u,t\right)}^{\text{load}}+p_{\left(u,t\right)}^{\text{PV}}$, where $p_{\left(u,t\right)}^{\text{PV}}\leq 0$. The net overproduction of the LEC available for AEV charging at MMR rates at each time step t is then given as:

$$OLEC_{\left(t\right)}=\max\left(0,\;-\sum_{u\in L}\lambda_{\left(u,t\right)}\right)$$

Positive values indicate surplus renewable energy available for AEV charging at preferential rates. As shown in Fig. 3, the overproduction profiles exhibit the expected diurnal pattern with nonzero values concentrated during midday hours (approximately 10:00–16:00). LEC B has substantially higher peak overproduction (≈19.2 kWh) compared to LEC A (≈6.7 kWh), reflecting differences in installed PV capacity relative to consumption within each community.

**Electricity tariff construction:** The electricity tariffs used in the data are taken from the SU Portuguese retailer.^1^ The time-of-use tariff follows the Portuguese tri-hourly retail structure with three price levels: off-peak (0.1072 €/kWh, night and early morning hours), mid-peak (0.1741 €/kWh, morning and late afternoon), and peak (0.2400 €/kWh, midday and early evening). When community overproduction is available at a given time period, the MMR price for AEV charging is reduced. This MMR price is computed as a weighted average of the internal community trading price and the grid tariff, where the weight reflects the share of energy that can be sourced from the community surplus [11,12]. The flat tariff uses a constant price of 0.1654 €/kWh across all periods, with the same community adjustment mechanism. As visible in Fig. 3, the effective tariff can drop significantly below the grid rate during periods of high solar overproduction, creating incentives for algorithms to schedule charging during these windows.

**Instance generation and scaling:** Instances were generated for 10 size configurations: (K, N) = (2, 20), (4, 40), (6, 60), (8, 80), (10, 100), (12, 120), (14, 140), (16, 160), (18, 180), (20, 200). For each configuration, two tariff variants were produced, yielding a total of 20 instance files. The depot for all vehicles is located at the centroid of all request coordinates (approximately 41.167°N, 8.621°W). Each vehicle has a dedicated origin and end depot node (with identical coordinates) to allow flexible modeling of return-to-depot constraints. The energy community data, charging station locations, and overproduction profiles are identical across all instance sizes, as they represent the same physical communities. The instance files, travel time matrices, overproduction profiles, and MMR pricing vectors are all original outputs produced by the authors. Similarly, the LEC formation, centroid-based station placement, and prosumer clustering results are original contributions not available from any existing source. The full instance generation pipeline is implemented in the Julia script generate_instances.jl, and the LEC formation procedure is implemented in the Python script group_prosumers.py, both available in the Zenodo repository. The entire pipeline from raw input files to final instance files is fully automated by the released scripts, with no manual intervention required beyond specifying the input file paths.

## Limitations

This dataset should be interpreted as a reproducible benchmark rather than a complete operational digital twin. Its realism comes from the use of Porto taxi trajectories, OpenStreetMap-based travel times, prosumer-level load and PV profiles, and Portuguese electricity tariffs. However, the dataset presents some limitations users should consider when applying the proposed benchmarks, such as:

• The user requests are based on historical taxi trip origins and destinations in the city of Porto, Portugal, which constrain the geographical scope to a single urban environment with a specific road network topology, trip distance distributions, and spatial demand patterns. Researchers applying these instances should be aware that algorithmic performance may differ in cities with different urban layouts, traffic patterns, or demand densities.
• The pickup and dropoff time windows are synthetically generated by uniformly sampling the 24-hour scheduling horizon and assigning a fixed 30-minute width, rather than reflecting real passenger booking preferences or observed demand peaks. This design choice ensures full-day coverage and avoids bias toward particular time periods, but does not capture the concentration of demand during morning and evening peak hours typical of real dial-a-ride services.
• The electricity tariffs are taken from the Portuguese retailer SU Eletricidade and reflect the Portuguese tri-hourly time-of-use structure and flat tariff options available at the time of data collection. These tariff structures are specific to the Portuguese regulatory context and may differ substantially from tariff designs in other countries or future regulatory frameworks. The flat tariff variant is included precisely to allow researchers to investigate the sensitivity of routing decisions to tariff structure, and the LEC energy data workbook provides the full prosumer-level profiles needed to construct alternative tariff scenarios.
• The energy community profiles are based on a single day, without accounting for uncertainty for PV generation, and load consumption, or multi-day operational effects.
• Charging stations are placed at the geographic centroid of each community rather than at existing infrastructure locations, which may not reflect real-world constraints such as grid connection availability, land use, or installation costs. The instance file format is not restricted to the current configuration of two charging stations, the number of charging stations field in the header and the corresponding node and charging station blocks are fully parameterized, and can be modified in the instance generation file, and the group_prosumers.py script, which implements an alternative placement function that assigns charging station sites to each LEC, providing a ready-made mechanism for generating instances with infrastructure-constrained station placement.
• The AEVs are assumed to be homogeneous, meaning that they have the same battery capacity, consumption rate and passenger capacity, based on the characteristics of the Tesla Model 3 vehicle.

Future directions for this dataset could address several of the limitations presented, such as incorporating taxi or ride-hailing data from additional cities to broaden the geographic scope and enable cross-city algorithmic comparisons. Deriving time windows from real booking timestamps rather than uniform random sampling would increase the realism of demand. Additional seasonal or stochastic energy profiles would support research on robust and chance-constrained optimization under photovoltaic uncertainty. Heterogeneous fleet configurations with mixed battery technologies or vehicle types, as well as larger instance sizes beyond 200 requests, would extend the dataset's utility for scalable decomposition and learning-based methods. Finally, vehicle-to-grid (V2G) scenarios, in which AEVs discharge surplus energy back to the community during low-demand periods, represent a natural and timely extension that the existing LEC energy data workbook is already structured to support.

## Ethics Statement

This work does not involve human subjects, animal experiments, or data collected from individuals. The taxi trajectory data used to generate request locations is publicly available and fully anonymized, along with the community data, which had labels and descriptions removed, and the user locations were synthetically generated with no real user locations. No ethical approval was required.

## Credit Author Statement

**José Almeida:** Conceptualization, Methodology, Software, Validation, Formal analysis, Investigation, Data Curation, Writing – Original Draft, Writing – Review & Editing, Visualization. **Steffen Limmer:** Conceptualization, Methodology, Software, Investigation, Writing – Review & Editing. **João Soares:** Conceptualization, Validation, Resources, Writing – Review & Editing, Supervision, Funding acquisition. **Maria Bresich:** Methodology, Software, Validation, Writing – Review & Editing. **Günther Raidl:** Resources, Validation, Writing - Review & Editing. **Zita Vale:** Resources, Project administration, Funding acquisition.

## Data Availability

ZenodoDataset of Electric Autonomous Dial-a-Ride Instances with Local Energy Communities and Electricity Tariffs (Original data)

## Footnotes

1 https://sueletricidade.pt/ (Accessed 11 April 2026).
